# Thyme essential oil potentials as a bactericidal and biofilm-preventive agent against prevalent bacterial pathogens

**DOI:** 10.1038/s41598-025-16485-5

**Published:** 2025-08-27

**Authors:** Hayam M. Fathy, Marwa N. Ahmed, Hanan A. Goda, Mohamed A. Moselhy

**Affiliations:** Microbiology Department, Faculty of Agriculture, Cairo University, Giza, 12613 Egypt

**Keywords:** Pathogenic bacteria, Antibacterial activity, Biofilm, Thyme essential oil, Chemical composition, Molecular docking, Gene expression, Biotechnology, Microbiology, Molecular biology

## Abstract

Antimicrobial resistance represents a significant global issue that requires the investigation of innovative approaches for infection management. In pursuit of alternative natural antimicrobials, nine plant essential oils were evaluated for their antibacterial properties against nine common bacterial pathogens. Among the tested essential oils, thyme essential oil demonstrated the highest antibacterial activity against all tested bacterial species, Thyme essential oil exhibited inhibition zones ranging from 17.3 to 51 mm with relative minimum inhibitory concentrations ranging from 99.2 to 450 µg/ml, implying the bactericidal effect. The ultrastructural changes in bacterial cells treated with thyme essential oil were visualized using transmission electron microscope. Thyme essential oil exhibited a potent inhibitory effect toward the biofilm formations for all the tested pathogenic strains. GC/MS analysis was used to determine the thyme essential oil composition. The major components of thyme essential oil were thymol (28.29%), o-cymene (18.31%), ç-terpinene (8.51%), eucalyptol (5%), linalool (2.86%), borneol (2.17%), á-myrcene (1.55%), à-pinene (1.52%) and camphene (1%). Molecular docking analysis demonstrated that the constituents present in the thyme essential oil had high binding affinity for ECF, FimH, LasR, PrfA and RhlA proteins, which were found to be associated with improved anti-biofilm efficacy. Furthermore, treatment with thyme essential oil led to the downregulation of essential genes associated with virulence and biofilm formation in the tested pathogens. These findings suggest that thyme essential oil has promising potential as an antibacterial and a biofilm inhibitory agent to combat bacterial infections in food and pharmaceutical industries.

## Introduction

Antimicrobial resistance is a global public health issue that reduces the likelihood of effective treatment for infectious diseases. Over the past few decades, the overuse, and misuse of antimicrobial drugs for treatment or as growth-promoting agents in intensive husbandry have been major contributors to the rise in antibiotic resistance^1^. In this context, the capacity of bacteria to establish biofilms is a critical factor in bacterial resistance. A biofilm is a group of microorganisms embedded within a matrix that adheres to both biotic and abiotic surfaces. The bacterial cells within biofilms are safeguarded by the extracellular matrix composed of proteins, polysaccharides (EPS), and nucleic acids. Consequently, these bacterial cells demonstrate greater resistance to antibacterial agents compared to planktonic cells^2^. As a result, biofilm formation is one of the main barriers to effective antibiotic therapy. Previous studies indicate that the resistance exhibited by biofilm-associated cells is influenced by multiple factors, such as the existence of extracellular polymeric substances in the biofilm and the regulation of resistance gene expression^3^. As well, quorum sensing (QS), a bacterial communication system that uses diffusible molecules called autoinducers, is critical for inducing gene expression to regulate cell behaviors such as bioluminescence, the secretion of virulence factors, biofilm formation, and resistance to antimicrobial agents^4^.

Public health issues related to foodborne illnesses affect millions of individuals worldwide each year. Pathogenic bacteria such as *Escherichia coli* O157:H7, Methicillin-resistant *Staphylococcus aureus* (MRSA), *Listeria monocytogenes*, *Klebsiella pneumoniae*, *Bacillus cereus*, *Salmonella typhimurium* and *Campylobacter* sp., are responsible for a wide range of food related infections^5^. In addition, *Pseudomonas aeruginosa* is widely distributed in the environment and has emerged as a common causative agent of foodborne infections. Its ability to thrive in diverse food matrices through biofilm formation and resist various preservation methods makes it an important foodborne pathogen of significant public health concern^6,7^. Moreover, there is a growing interest in using natural antibacterial agents driven by consumer concerns regarding the safety of synthetic chemical preservatives in food products. In this context, the application of certain natural products derived from various plant sources presents a promising approach to combat the antimicrobial resistance and biofilm formation^5^.

Plant essential oils (EOs) are intricate natural liquids characterized by unique aromas and flavors, which are influenced by their specific chemical composition. EOs encompass various classes of compounds, mainly phenolic substances and terpenes such as monoterpenoids and sesquiterpenoids, along with aromatic compounds (phenylpropanoids, aldehydes, alcohols, esters) and aliphatic compounds (alkanes, aldehydes, alcohols, ketones, esters)^8^. EOs exhibit a wide range of activities due to their chemical complexity and diverse mechanisms of action. Considering their low molecular weight and lipophilic features, EOs can penetrate tissues rapidly and efficiently^9^. Therefore, they have been employed as anti-inflammatory, antioxidant, antimicrobial, antispasmodic, carminative, and simulative agents^10^. Herbs such as thyme, peppermint, lavender, basil, and sage, members of the *Lamiaceae* family, have been widely studied, with numerous *in vitro* investigations demonstrating the effectiveness of their EOs in combating prevalent foodborne pathogens^5,11,12^. Certain constituents of EOs interact with the lipid bilayer of bacterial cell membranes, while others disrupt the bacterial cell cycle or impede processes such as protein synthesis and DNA replication, inhibiting bacterial motility, blocking QS activity, preventing cell adhesion and reducing EPS formation^13^. Various hypotheses have been proposed regarding the antimicrobial properties whether bactericidal or bacteriostatic of individual EO components. Furthermore, certain constituents (e.g., thymol, carvacrol, β-caryophyllene, pinene and cymene) may exhibit stronger antimicrobial effects than others^14^.

Despite extensive research on the antimicrobial effects of EOs, many previous studies have primarily focused on general antibacterial activity or basic phenotypic assays, with limited exploration of their precise molecular targets and mechanistic pathways. Moreover, comprehensive assessments integrating ultrastructural observations, chemical profiling, *in silico* predictions, and gene-level effects remain scarce. In particular, the molecular mechanisms underlying the anti-biofilm efficacy of thyme essential oil (TEO), including its interaction with specific biofilm-associated proteins and gene regulation pathways, are not fully understood. This study addresses this gap by combining phenotypic assays with transmission electron microscopy (TEM), GC/MS analysis, molecular docking, and gene expression profiling to offer a holistic understanding of TEO antibacterial and antibiofilm mechanisms.

Therefore, this study aimed to evaluate the antibacterial activity of nine EOs against nine common bacterial pathogens. The chemical profile of the most potent EO was determined, and its potential to inhibit bacterial biofilm formation was assessed. Molecular docking analyses were conducted to investigate the interactions between the EO components and key biofilm-associated proteins. In addition, the efficacy of the most active EO was examined by evaluating its impact on the expression of virulence and biofilm-associated genes.

## Materials and methods

### Essential oils

The EOs used in this study were ginger oil, basil oil, thyme oil, peppermint oil, clove oil, sage oil, geranium oil, lavender oil, and garlic oil. All tested EOs were purchased from Essential Oils Laboratory, National research center (NRC), Giza, Egypt. All EOs were extracted by the steam distillation method and stored in the dark bottles at 0–4 °C until further use.

### Determination of antimicrobial activity of essential oils

#### Target microorganisms

The pathogenic bacterial strains used in this study were obtained from the American Type Culture Collection (ATCC). These include *Staphylococcus aureus* ATCC 25,923, Methicillin-resistant *Staphylococcus aureus* (MRSA) ATCC 43,300, *Bacillus cereus* ATCC 33,018, *Listeria innocua* ATCC 33,090 and *Listeria monocytogenes* ATCC 19,115 as Gram-positive bacteria (G+), while *Pseudomonas aeruginosa* ATCC 35,032, *Salmonella enterica* subsp. *enterica* serovar Typhimurium ATCC 14,028, Shiga toxin-producing *Escherichia coli* (STEC) wild type strain 93111, and *Escherichia coli* O157:H7 ATCC 700,728 as Gram-negative bacteria (G^_^). All bacterial strains were cultured overnight at their optimum temperatures in tryptone glucose yeast extract (TGY) broth medium.

#### Agar well-diffusion assay

The antimicrobial properties of EOs were evaluated by agar well diffusion method as described by Hassan et al.^15^. Petri dishes containing solid Miller-Hinton (M.H.) agar medium were seeded with 100 µl of bacterial broth culture (10^8^ CFU/ml, 0.5 McFarland standard). Then 6.0 mm diameter wells were punched into agar using a sterile corkborer. The tested EOs were applied in a concentration of 100 mg/ml in dimethyl sulfoxide (DMSO) and 60 µl of each oil were transferred into the wells in triplicates. The control wells containing DMSO were used as negative control. Discs loaded with 10 µg of gentamicin and ampicillin were served as positive standard control for Gram-positive and Gram-negative bacteria, respectively. The plates were incubated at optimum temperatures (30–37 °C) for 24 h. Antimicrobial activity was estimated by measuring the diameter of the zone of inhibition.

#### Determination of minimum inhibitory concentration (MIC) and minimum bactericidal concentration (MBC)

The MIC values of potent EOs against tested bacteria were determined using broth dilution method as reported by Andrews^16^. Trials were carried out on 96-well microplates. Two-fold serial dilutions of each oil were prepared from a stock solution (1600 µg/ ml) in M.H. broth medium (100 µl) supplemented with DMSO. Then 100 µl from each fresh bacterial broth culture (containing 10^8^ CFU/ml) was inoculated in the prepared concentrations of EOs. Preparations were incubated at optimum temperatures (30–37 °C) for 24 h. The negative control was M.H. broth medium supplemented with DMSO and oil, positive control was bacterial cultures in M.H. broth supplemented with DMSO without oil. The bacterial growth (OD) was measured at 480 to 520 nm. The lowest concentration of the oil that had no visible bacterial growth was considered as MIC. Then, the MBC was ascertained by subculturing the test dilutions that exhibited no observable bacterial proliferation onto the M.H. agar medium, which is devoid of oils, and subsequently incubated for a duration of 18–24 h at the optimal temperature. The lowest concentration that did not yield any individual bacterial colony on the solid medium was designated as the MBC. All experimental treatments were conducted in triplicate.

### Transmission electron microscopy (TEM) analysis

The morphological and ultrastructure changes of *L. monocytogenes*, *S. aureus*, *S. aureus* MRSA, *B. cereus*, *E. coli* O157:H7, Shiga toxin-producing *E. coli* (STEC) and *S. typhimurium* due to the treatment of TEO were assessed using TEM^17^. The effect of geranium oil on *P. aeruginosa* was also tested. To accomplish the TEM analysis, each bacterial strain was grown in M.H. broth medium supplemented with the tested oil in DMSO (at a concentration of MIC) and incubated at the optimum temperatures (30–37 °C) for 24 h. The control cultures were prepared in M.H. broth medium free of oil. After incubation, the cultures were centrifuged at 4000 rpm for 10 min at the room temperature. The collected cells were washed three times with phosphate buffer (PB), fixed in glutaraldehyde and osmium tetroxide, dehydrated in ascending series of ethanol (40, 50, 60, 70, 80, 90, 95, and 100%) for 15 min each and embedded in an epoxy resin. The Leica Ultracut UCT ultramicrotome (Leica, Wetzlar, Germany) was used to prepare ultra-thin microtome sections to be examined by transmission electron microscope JEOL (JEM-1400 TEM, Tokyo, Japan) at the candidate magnification. Images were captured by CCD camera model AMT, Optronics camera with 1632 × 1632-pixel format as side mount configuration.

### Biofilm formation assay

The effect of TEO on biofilm inhibition was assessed for all tested bacterial strains except *L. innocua* using the crystal violet assay as described by Santiago et al.^18^. Pathogenic bacterial strains were cultured overnight in LB broth at 37 °C for 18 h, except for *B. cereus*, which was incubated at 30 °C. The cultures were then diluted to an OD of 0.1 at 600 nm in fresh LB. TEO was prepared in two-fold serial dilutions, ranging from a concentration of 425 to 7 µg/ml, using LB broth. A 96-well polystyrene microtiter plate was used for the assay, each well containing 100 µl of diluted TEO and 100 µl of bacterial suspension. Blank wells contained LB without TEO and bacteria, while control wells contained bacterial suspensions without any treatment. The plate was incubated at 37 °C for 48 h to allow biofilm formation, except for *B. cereus*, which was incubated at 30 °C. After incubation, the planktonic cells were removed by gently washing the wells with sterile phosphate-buffered saline (PBS). Biofilms were stained by adding 200 µl of 0.3% crystal violet solution to each well and incubating for 20 min at room temperature. Excess crystal violet was removed by washing the wells three times with distilled water. The plates were allowed to dry for 1 h at room temperature. The bound crystal violet was then solubilized by adding 200 µl of ethanol 95% for 30 min at room temperature. The absorbance was measured at 570 nm by microplate reader (BioRad Laboratories Inc., model 3350, Hercules, California, USA) using ethanol 95% as the blank. The percentage of biofilm inhibition was calculated based on the reduction in absorbance compared to the positive control wells. Six replicates were assigned for controls and for each oil dilution.

### Gas chromatography mass spectrometry (GC/MS) analyses

The chemical composition of TEO was analyzed using Trace GC-TSQ mass spectrometer (Thermo Scientific, Austin, TX, USA) under recommended conditions^9^. A DB-5 capillary column (ZB-5HTMS; comprising 5% diphenyl and 95% dimethylpolysiloxane) characterized by dimensions of 30 × 0.25 mm inner diameter and a film thickness of 0.25 μm was utilized as the stationary phase. The gas chromatography (GC) temperature regimen commenced with an initial isothermal period at 40 °C for a duration of 2 min, succeeded by a temperature elevation at a rate of 10 °C per minute until reaching 250 °C. Following this, the temperature was further escalated at a rate of 20 °C per minute to attain 280 °C, where it was maintained for an additional 10 min. For the gas chromatography-mass spectrometry (GC-MS) detection, an electron ionization apparatus was employed, functioning in electron impact mode with an ionization energy established at 70 eV. Helium (99.99%) was utilized as the carrier gas, maintained at a consistent flow rate of 1 ml/min, and a sample injection volume of 1 µl was applied. The injector temperature was consistently upheld at 250 °C, whereas the ion-source temperature was calibrated to 220 °C. Mass spectral data were acquired at 70 eV, with a scanning interval set at 1 s, capturing fragment ions with mass-to-charge ratios ranging from 50 to 4500 Da. The components were identified by comparison of their mass spectra with those of WILEY 09 and NIST 14 mass spectral database.

### Molecular docking analysis

Molecular Operating Environment (MOE) software was utilized to evaluate the binding potential of specific compounds from TEO, including o-cymene, c-terpinene, caryophyllene, and phenol, 5-Methyl-2-(1-methylethyl), against key proteins involved in biofilm formation across different bacterial species^19,20^. The protein targets analyzed were chosen based on their documented roles in biofilm structure, adherence, and regulatory functions, including RhlA and LasR from *P. aeruginosa*^21^ FimH from *E. coli*^22^the ECF transporter from *S. aureus*^23^and PrfA from *L. monocytogenes*^24^. These proteins were sourced from the Protein Data Bank (PDB) using the following identifiers: PrfA (5F1R), RhlA (8IK2), LasR (4NG2), FimH (5JCR), and the ECF transporter (3P5N). Each protein structure underwent preliminary preparation using MOE, where hydrogen atoms were added, protonation states were adjusted to simulate physiological pH conditions, and the structures were energy minimized to ensure stability. This was done to avoid any conformational anomalies that might affect docking accuracy. The four TEO compounds: o-cymene, c-terpinene, caryophyllene, and 5-Methyl-2-(1-methylethyl) phenol- were modeled using the molecular builder tool within MOE. These compounds were then energy minimized using the MMFF94x force field to obtain the lowest energy conformations, representing their bioactive forms. To mimic *in vivo* conditions, the compounds were also protonated at a physiological pH of 7.4 before being subjected to docking. Using MOE’s docking tool, the prepared compounds were docked onto the active sites of each target protein. Standard docking protocols were applied, and the scoring function within MOE was used to rank the binding poses based on interaction strength. For each protein-compound complex, the interactions were analyzed, with particular attention given to hydrogen bonding, hydrophobic interactions, and π-π stacking where applicable. The highest-ranking poses with optimal binding interactions were selected for further analysis to understand the molecular basis of their inhibitory effects on biofilm-related functions.

### Gene expression analysis

Total RNA was extracted from both 0.5 MIC-TEO treated *E. coli* O157:H7 and *S. aureus* as well as untreated control cells using the GeneDireX RNA extraction kit (Taiwan), following the manufacturer’s protocol. The RNA concentration and purity were assessed using a NanoDrop spectrophotometer. To ensure RNA integrity, 1% agarose gel electrophoresis was performed. Subsequently, 1 µg of RNA was used for cDNA synthesis with Xpert cDNA synthesis kit (Grisp), according to the kit instructions. Gene expression analysis was performed using SYBR Green master mix (Grisp) in conjunction with ROX dye for normalization. The reactions were carried out in a StepOnePlus™ Real-Time PCR System (Applied Biosystems, USA). Each reaction contains 10 µL of SYBR Green master mix, 1 µl of diluted cDNA, 0.5 µl of forward and reverse primers (specific for the genes being analyzed, and the 16S rRNA reference gene), and nuclease-free water to a total volume of 20 µl. The cycling conditions were as follows: initial denaturation at 95 °C for 10 min, followed by 40 cycles of 95 °C for 15 s, and 60 °C for 30 s. The relative expression of *stx1*,* stx2*,* csgA*,* fimH*,* icaA*, and *icaD* was calculated using the 2^(-ΔΔCt) method^25^with the 16S rRNA gene serving as the internal reference for normalization. The genes *stx1* and *stx2*, encoding Shiga toxins as well as *csgA* and *fimH*, associated with biofilm formation, were analyzed in *E. coli* O157:H7, respectively. While, in *S. aureus*, the biofilm-related genes *icaA* and *icaD* were examined. The primer sequences of the selected genes are listed in Table 1. All samples were performed in triplicate.

**Table 1:** Primer sequences for real-time PCR.

Gene name	Target organism	Primer sequence (5’→3’)
*stx1*	*E. coli* O157:H7	Forward: ACACTGGATGATCTCAGTGG Reverse: CTGAATCCCCCTCCATTATG
*stx2*	*E. coli* O157:H7	Forward: CCATGACAACGGACAGCAGTT Reverse: CCTGTCAACTGAGCAGCACTTTG
*csgA*	*E. coli* O157:H7	Forward: AAGCTTGATAACAGCGTATTTACGTGGG Reverse: GGATCCCAACTTCGTCAAAGCAATGGG
*fimH*	*E. coli* O157:H7	Forward: GTGCCAATTCCTCTTACCGTT Reverse: TGGAATAATCGTACCGTTGCG
*icaA*	*S. aureus* ATCC 25,923	Forward: AAAATCGATGGTAAAGGTTGGC Reverse: AGTTCTGCAGTACCGGATTTGC
*icaD*	*S. aureus* ATCC 25,923	Forward: ATGGTCAAGCCCAGACAGAG Reverse: AGTATTTTCAATGTTTAAAGCAA
16S rRNA	Universal	Forward: AGAGTTTGATCMTGGCTCAG Reverse: TACGGYTACCTTGTTACGACTT

### Statistical analysis

All experiments were carried out with a minimum of three replicates, and results were presented as mean ± standard deviation. The statistical analyses were done using R (version 3.2.5). Student’s t test was used to test the significance of comparisons. The differences were considered significant when the *P* value was ≤ 0.05.

## Results and discussion

### Antibacterial potential of the tested EOs

The antimicrobial properties of nine EOs were assessed against nine pathogenic bacterial strains using the well-diffusion assay method. Table 1 represents the inhibition zone values of the susceptibility test. *L. innocua* showed resistance to most of the tested EOs and even to the standard antibiotic control. Interestingly, TEO exhibited a remarkable effect against *L. innocua* (inhibition zone of 28 mm). Consistently, TEO exhibited superior values for inhibition zones for all tested G + bacterial strains (23.7–51 mm) that surpassed the other tested EOs (8.7–38 mm) and the standard control antibiotic (12–19 mm). Likewise, TEO showed the highest inhibition activities against G^_^ bacteria ranged from 17.3 to 49.3 mm compared to the other EOs (6 to 25 mm) and the control antibiotic (15 to 20 mm). However, garlic and sage EOs demonstrated less efficacy on the tested bacteria. Many plants’ EOs have been tested for their antimicrobial activities. In the present work, out of nine EOs, TEO possessed substantial antibacterial properties. These results are consistent with those of Aljabeili et al.^26^ who reported the *in vitro* antibacterial activity of TEO against G + bacteria (30–38 mm) and G^_^ bacteria (25–31 mm) using agar disk-diffusion technique. However, mixing chitosan (2%) with pure TEO reduced this efficacy. Comparable inhibition zone values were also reported by Veloso et al.^27^ for G + bacteria (40–46.5 mm) and G^_^ bacteria (37–39 mm) due to the treatment with TEO. In another study, Ozogul et al.^28^ suggested that utilization of nanoemulsions based on TEO enhanced the inhibition of foodborne bacterial pathogens. Kryvtsova et al.^29^ documented even higher inhibition zones (45–65 mm) against clinical and typical *S. aureus* strains. Although our results align with these studies, they serve to complement rather than validate previous findings, given differences in bacterial strains, EO formulations, and testing conditions.

The current results also support the notion that the EOs from different thyme species showed a remarkable selectivity towards G + bacteria and moderate effect against G^_^ bacteria^30^. However, Imelouane et al.^31^ found that the tested G^_^ bacteria were more sensitive to the TEO than Gram + bacteria. A significant portion of the antimicrobial efficacy of TEO seems to be linked with phenolic and terpene constituents^32^. EO components, particularly phenolic derivatives, disrupt the lipid composition of the bilayer membrane and possess the ability to interact with cellular organelles, thereby inducing antibacterial effects^33^.

### MIC and MBC of essential oils

The agar diffusion methodology is regarded as a preliminary evaluative approach for the antibacterial efficacy of the substances under examination, providing a foundational indication for subsequent quantitative evaluations of the MIC and MBC^34^. The results of MIC and MBC analyses are shown in Tables 3 and 4. The antimicrobial efficacy of the tested EOs, assessed through the MIC and MBC, exhibited a consistent relationship with the diameters of the inhibition zones (Table 2); the results revealed that TEO displayed extensive antimicrobial potency against all tested bacterial strains except for *P. aeruginosa* in which geranium essential oil (GEO) was the most efficient treatment (Table 3). The essential oils’ MICs were categorized using the standards established forward by numerous authors as high (MIC < 600 µg/ml), moderate (MIC from 600 to 2500 µg/ml), low (MIC > 2500 µg/ml)^35,36^. Hence, the MIC analysis of this study for TEO and GEO showed a high antibacterial activity against all tested pathogenic strains. The MIC values for both EOs ranged from 99.2 to 450 µg/ml. By comparing the sensitivity of the tested bacterial strains to TEO, *B. cereus* and *E. coli* O157 STEC were more sensitive, while *L. innocua* demonstrated the highest resistance. Veloso et al.^27^ reported comparable MIC values for TEO (75–620 µg/ml) for five food born bacterial pathogens. However, Boskovic et al.^37^ and Aljabeili et al.^26^ documented higher MIC values for TEO, ranging from 320 to 640 µg/ml and 40–270 mg/ml, respectively. The antimicrobial efficacy of TEO is contingent upon its chemical constituents. The synergistic interactions among bioactive compounds are accountable for the distinct mechanisms of antimicrobial action. According to Kang et al.^38^ and Veloso et al.^27^ TEO is characterized by a high concentration of *p*-cymene, carvacrol, thymol, and γ-terpinene that demonstrated a capacity to impede the proliferation of G + bacteria through mechanisms that include membrane disruption, modifications in cellular morphology, and a reduction in the intracellular ATP reservoir. The hyper-permeabilization of the bacterial cytoplasmic membrane, which leads to a diminishment of membrane potential, the failure of proton pumps, and a depletion of ATP, potentially represents the primary mechanism through which TEO exerts its antimicrobial effects against G^_^ bacteria.

In the present study, the MBC values of TEO and GEO were ≤ 4 times of MIC values (Table 4), suggesting their applications as strong natural bactericidal substances. Dejoies et al.^39^ and Moselhy et al.^40^ mentioned that the effectiveness of an antibacterial agent is primarily determined by the MBC/MIC ratio. Thus, an antibacterial agent is classified as bactericidal when the MBC value does not exceed four times the MIC value. The results of this study are in agreement with the findings of Drioiche et al.^30^who observed that the values of MBC was equal or more than the values of MIC for TEO against the tested pathogens.

According to the aforementioned results, TEO was selected for further investigations to evaluate its bactericidal potency against all tested pathogenic bacteria except in the case of *P. aeruginosa*, GEO was used.

**Table 2:** The antibacterial activities of nine essential oils against tested bacterial strains.

Strain	Inhibition zone of essential oils (mm)									
	Ginger	Basil	Thyme	Peppermint	Clove	Sage	Garlic	Geranium	Lavender	Positive control^*^
*L. monocytogenes* ATCC 7644	12.3 ± 0.58	10.7 ± 0.58	51.0 ± 1.7	15.0 ± 0.0	13.7 ± 0.58	R	R	13 ± 0.0	10 ± 0.0	19.0 ± 0.0
*L. innocua* ATCC 33,090	R	R	28.0 ± 2.65	R	R	R	R	R	R	R
*S. aureus* MRSA ATCC 43,300	10.0 ± 0.0	10.7 ± 0.58	23.7 ± 0.58	15.7 ± 0.58	R	R	R	8.7 ± 0.58	9.7 ± 0.58	12.0 ± 1.0
*S. aureus* ATCC 25,923	10.0 ± 0.0	11.7 ± 1.15	27.0 ± 2.65	15.7 ± 0.58	13.7 ± 0.58	R	R	14 ± 0.0	11.0 ± 0.0	16.0 ± 1.0
*B. cereus* ATCC 33,018	38.0 ± 0.0	17.0 ± 0.0	37.0 ± 0.0	15.0 ± 0.3	19.0 ± 0.0	10.0 ± 0.0	12.0 ± 0.0	10 ± 0.0	11.3 ± 0.58	19.0 ± 1.0
*E. coli* O157 STEC 93,111	25.0 ± 0.0	10.0 ± 0.0	23 ± 0.0	17.0 ± 0.0	R	10.0 ± 0.0	R	10 ± 0.0	12 ± 0.0	R
*E. coli* O157:H7 ATCC 700,728	6.0 ± 0.0	8.0 ± 0.0	30.0 ± 0.0	10.0 ± 0.0	12.0 ± 0.0	15.0 ± 0.0	10.0 ± 0.0	10.3 ± 0.58	10.7 ± 1.15	17.0 ± 0.0
*P. aeruginosa* ATCC 9027	15.3 ± 0.58	13.3 ± 0.58	17.3 ± 0.58	13.7 ± 0.58	17.0 ± 1.0	11.3 ± 0.58	8.3 ± 0.58	10 ± 0.0	9.0 ± 0.0	15.0 ± 0.0
*S. typhimurium* ATCC 14,028	10.7 ± 0.58	11.3 ± 0.58	49.3 ± 0.58	19.0 ± 1.0	16.3 ± 0.58	9.7 ± 0.58	R	11 ± 1.0	9.0 ± 0.0	20.0 ± 1.0

R: resistant, *: gentamicin (10 µg) for G + bacteria and ampicillin (10 µg) for G- bacteria, values are the mean ± SD.

**Table 3:** The MIC of the essential oils against tested bacterial strains.

Strain	MIC (µg/ml)								
	Ginger	Basil	Thyme	Peppermint	Clove	Sage	Garlic	Geranium	Lavender
*L. monocytogenes* ATCC 7644	879	899	198.4	941	271	R	R	298.5	298.5
*L. innocua* ATCC 33,090	R	R	450	R	R	R	R	R	R
*S. aureus* MRSA ATCC 43,300	296	450	198.4	317.6	R	R	R	380.8	402.1
*S. aureus* ATCC 25,923	439	312.4	198.4	317.6	370.6	R	R	754.6	321.3
*B. cereus* ATCC 33,018	230	312.4	99.2	196	241	332	432.3	450.5	181.1
*E. coli* O157 STEC 93,111	257.5	450	99.5	313.7	R	310.8	R	300.5	260.2
*E. coli* O157:H7 ATCC 700,728	439.5	324.8	204.9	580.8	280.3	455.4	1059	250.5	280.8
*P. aeruginosa* ATCC 9027	879	899	398	941	965	886	1059	288.6	474.6
*S. typhimurium* ATCC 14,028	879	274.8	198.4	267.6	270.6	443	R	330.3	349.2

R: resistant.

**Table 4:** The MIC and MBC of the most effective essential oils against tested bacteria.

Strain	Essential oil	MIC (µg/ml)	MBC (µg/ml)	MBC/MIC
*L. monocytogenes* ATCC 7644	Thyme oil	198.4	508	2.56
*L. innocua* ATCC 33,090	Thyme oil	450	1728	3.84
*S. aureus* MRSA ATCC 43,300	Thyme oil	198.4	794	4.0
*S. aureus* ATCC 25,923	Thyme oil	198.4	620	3.12
*B. cereus* ATCC 33,018	Thyme oil	99.2	332	3.35
*E. coli* O157 STEC 93,111	Thyme oil	99.5	99.5	1.0
*E. coli* O157:H7 ATCC 700,728	Thyme oil	204.9	410	2.0
*P. aeruginosa* ATCC 9027	Geranium oil	288.6	900	3.12
*S. typhimurium* ATCC 14,028	Thyme oil	198.4	381	1.92

### Transmission electron microscopy (TEM)

**Fig. 1:**
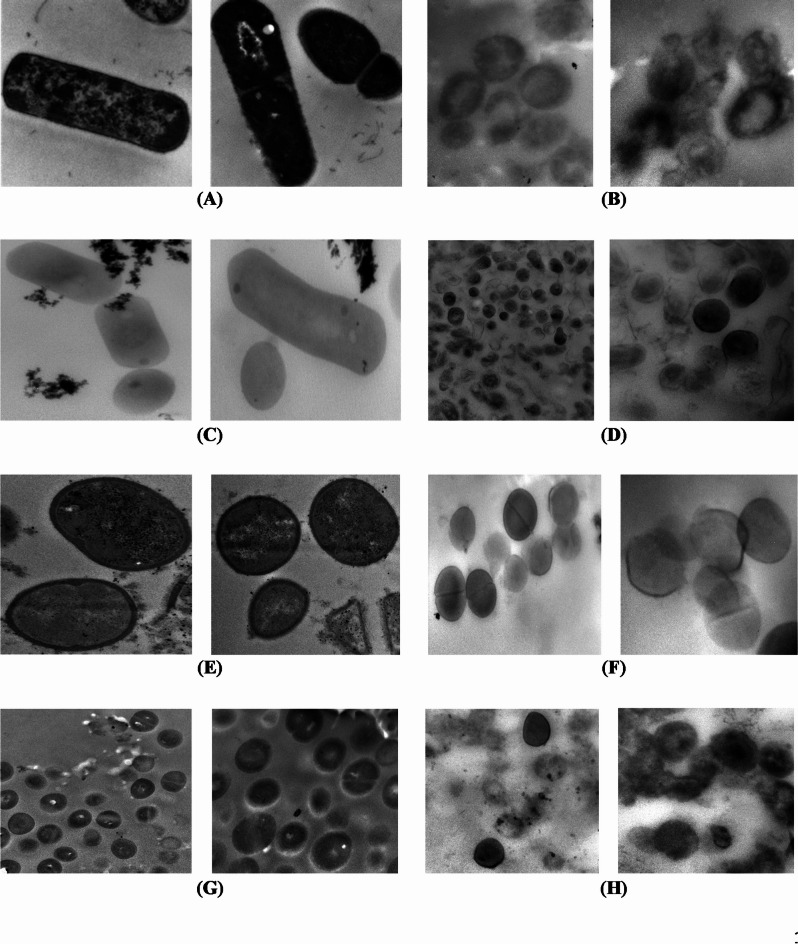
TEM micrographs of control and essential oil treated Gram-positive bacteria (*B. cereus*, *L*. *monocytogenes*, *S. aureus* and *S. aureus* MRSA). (**A**) Untreated *B. cereus* (Direct Mag.: 30,000×). (**B**) Treated *B. cereus* (Direct Mag.: 40,000×). (**C**) Untreated *L. monocytogenes* (Direct Mag.: 30,000×). (**D**) Treated *L. monocytogenes* (Direct Mag.: 15,000 and 40,000×). (**E**) Untreated *S. aureus* (Direct Mag.: 30,000×). (**F**) Treated *S. aureus* (Direct Mag.: 25,000 and 40,000×). (**G**) Untreated *S. aureus* MRSA (Direct Mag.: 15,000 and 30,000×). (**H**) Treated *S. aureus* MRSA (Direct Mag.: 25,000 and 30,000×).

**Fig. 2:**
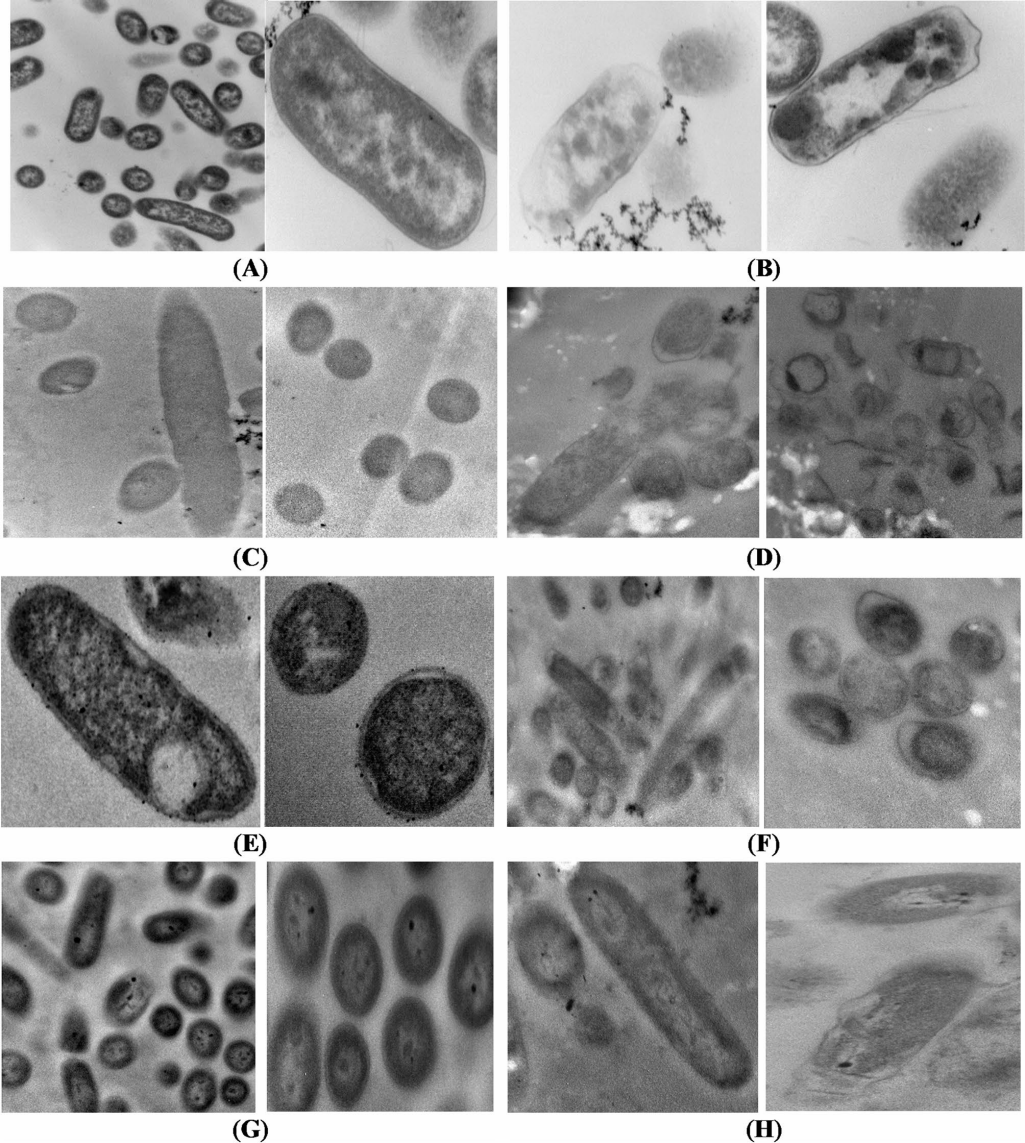
TEM micrographs of control and essential oil treated Gram-negative bacteria (*E. coli* (STEC), *E. coli* O157:H7, *S. typhimurium* and *P. aeruginosa*). (**A**) Untreated *E. coli* (STEC) (Direct Mag.: 10,000 and 40,000×). (**B**) Treated *E. coli* (STEC) (Direct Mag.: 30,000 and 40,000×). (**C**) Untreated *E. coli* O157:H7 (Direct Mag.: 12,000 and 15,000×). (**D**) Treated *E. coli* O157:H7 (Direct Mag.: 20,000×). (**E**) Untreated *S. typhimurium* (Direct Mag.: 20,000 and 60,000×). (**F**) Treated *S. typhimurium* (Direct Mag.: 15,000 and 25,000×). (**G**) Untreated *P. aeruginosa* (Direct Mag.: 15,000 and 30,000×). (**H**) Treated *P. aeruginosa* (Direct Mag.: 20,000 and 25,000×).

Transmission electron microscopy was employed to appraise the morphological and ultrastructure alterations of the bacterial cells treated with the most promising EOs having the lowest MIC. All tested bacterial cells, except *P. aeruginosa*, were treated with TEO. The cells of *P. aeruginosa* were treated with GEO. The micrographs of longitudinal and transverse sections of untreated cells indicated the regular cell morphology and homogenous cell components including cell wall, cell membrane, nuclear region, and ribosomes (Figs. 1 and 2). Also, the cell binary fission appeared in the cells of *B. cereus*, *S. aureus*, and *S. aureus* MRSA. The cleavage furrow was observed in *S. aureus* (Fig. 1E). In this stage, the FtsZ proteins, encoded by *ftsZ* gene, self – gather to form the Z ring around the division site between the two chromosomes. In the cells of *B. cereus* (Fig. 1A) and *S. aureus* MRSA (Fig. 1G), the formation of septum, which is directed by the Z ring, was shown. Generally, septum appears between the nucleoids, and extends gradually from the periphery toward the cell center to form two daughter cells^41^. The ultrastructural changes in oil treated bacteria are presented in Figs. 1 and 2 for G + and G^_^ bacteria, respectively.

For G + bacteria, the transverse sections of treated *B. cereus* (Fig. 1B) and *L. monocytogenes* (Fig. 1D) showed diverse degrees of cell lysis extending from partial cell lysis in the most cells to complete lysis in fewer cells allowing the leakage of cytoplasm and its components. *S. aureus* cells were less affected by TEO showing an effect of weak cell lysis only in some treated cells. Conversely, the other cells were healthy cells indicated by the formation of the septum. The complete cell lysis was exhibited in the *S. aureus* MRSA cells (Fig. 1H), and no visual effect was observed in some cells.

The TEM examination of treated G^_^ bacteria revealed the cell distortion and cell wall degradation in Shiga toxin-producing *E. coli* treated with TEO (Fig. 2B), and *P. aeruginosa* treated with GEO (Fig. 2H). The effects of cell distortion, partial and complete cell lysis were recorded in *E. coli* O157: H7 (non-STEC) (Fig. 2D). The TEM micrographs illustrated that the cells of *S. typhimurium* were less affected than *E. coli* to some extent (Fig. 2F). Only cell distortion and partial cell wall degradation were representative effects.

Generally, the TEM results confirmed that the antimicrobial activity of EOs is not assigned to a specific mechanism, but various mechanisms were described to demonstrate the activity against bacterial cells. This finding is compatible with Da Silva et al.^42^. Mainly, the antimicrobial effect of the essential oils depends on the interaction between hydrophobic components of the oil and lipids of the cell membrane. This interaction causes loss of membrane integrity, and this leads to cell membrane permeabilization resulting in ions and metabolites leakage, changes in absorption of nutrients, and in protein and nucleic acid synthesis. Also, the enzymes imperative for metabolism could be inhibited causing cell death^42,43^.

Also, the complete cell lysis of G + and G^_^ bacteria was observed. This indicates that the EOs could be active against bacteria through inhibition of cell wall synthesis. This inhibition could be attributed to interfering with the action of transglycosidase enzymes leading to preventing the insertion of new peptidoglycan monomers or attributed to the binding to transpeptidase enzyme leading to preventing cross-linkage of the two glycan linked peptide chains and autolysin releasing. In addition, degradation of the cell wall in G^_^ bacteria is accompanied by hydrolysis of the outer membrane which is related to the toxicity of pathogenic G- bacteria to humans. So, destruction of the outer membrane is associated with the loss of its toxicity to humans^41^.

### Biofilm Inhibition effect of TEO

**Fig. 3:**
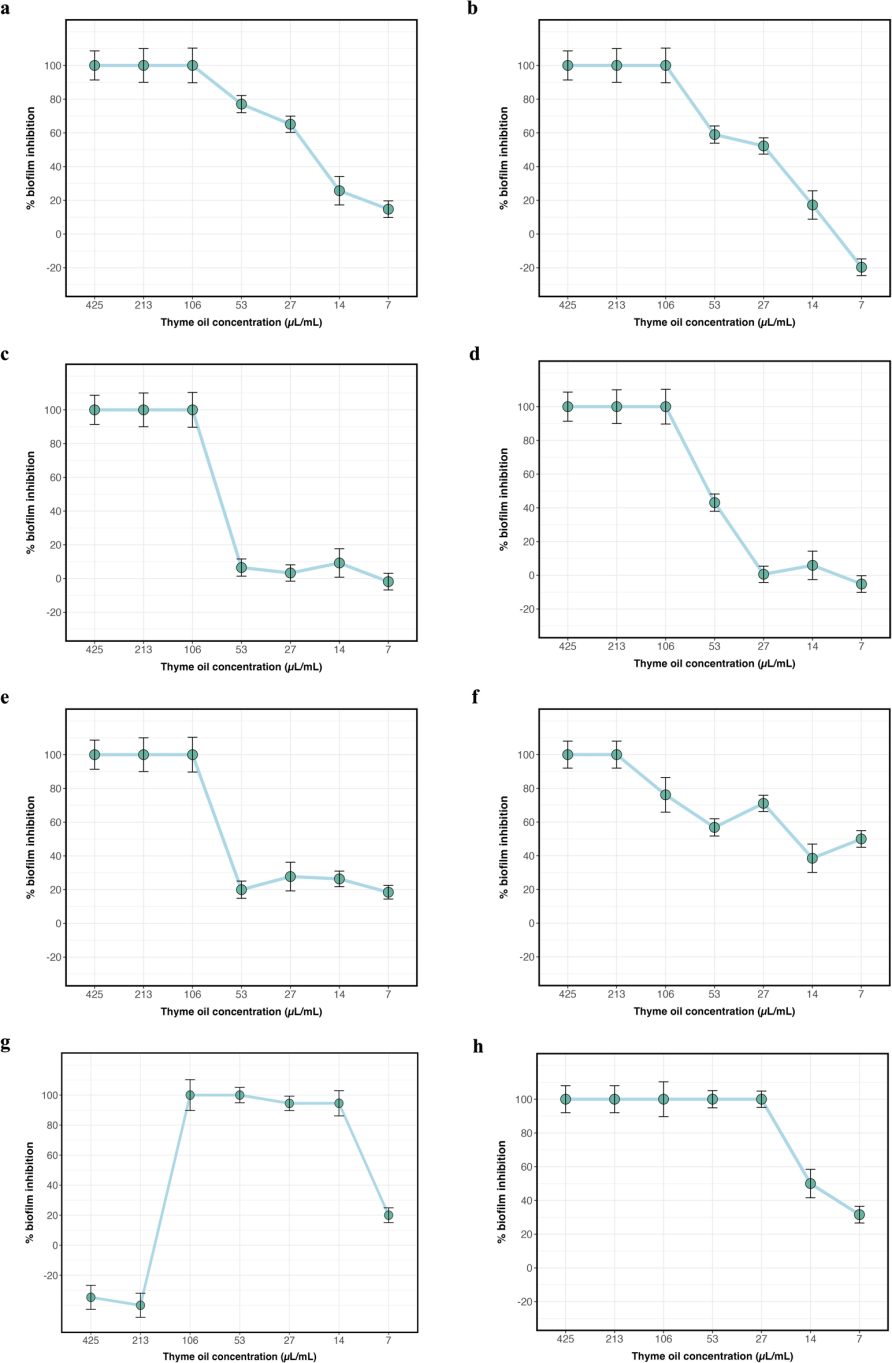
The impact of different concentrations of thyme essential oil on the inhibition of biofilm formation by pathogenic bacteria. (**a**) *S. aureus*, (**b**) *S. aureus* MRSA, (**c**) *B. cereus*, (**d**) *E. coli* (STEC), (**e**) *L. monocytogenes*, (**f**) *S. typhimurium*, (**g**) *E. coli* O157:H7, (**h**) *P. aeruginosa*. Data represents the mean of six replicates. Error bars represent standard deviation.

The crystal violet assay was used to assess the effect of TEO on the inhibition of biofilm formation by a number of G + and G^_^ pathogenic bacteria. In this study, biofilm formation of eight pathogenic bacterial strains was allowed to take place in the presence of augmented concentrations of TEO. Remarkably, TEO exhibited a potent inhibitory effect toward the biofilm formations for all the tested pathogenic strains (Fig. 3). The three highest concentrations (425, 213, and 106 µg/ml) of TEO exhibited 100% inhibition of biofilm formation for the majority of tested pathogenic bacteria, and the effect started to decline gradually by lowering the concentrations. Intriguingly, the high concentrations of TEO did not cause any observable effect on *E. coli* O157: H7 biofilm formation.

However, lower concentrations (106, 53, 27, and 14 µg/ml) of TEO resulted in 100% inhibition of biofilm formation. This paradoxical effect is known as the “Eagle effect” demonstrated with some antimicrobials where higher concentrations of an applied antimicrobial agent become less effective^44^.

These findings indicate the complexity of the interaction of antimicrobials with bacterial biofilms and that the optimal inhibiting concentrations applied toward the biofilm may vary among different bacterial species, thus requiring an optimized and tailored approach to apply effectively in a therapeutic manner. Various mechanisms have been demonstrated regarding the anti-biofilm effects of TEO, which is mainly attributed to its high content of bioactive compounds, especially thymol and carvacrol. Among the most important features of TEO is the inhibition of quorum sensing, the process of cell-to-cell communication required for the development and persistence of bacterial biofilms. The disruption of these signaling pathways by TEO would not enable the bacteria to coordinate biofilm growth and architecture, thus impairing the structural integrity and survival of the biofilm^45,46^. Another important mechanism is the disruption of the bacterial cell membrane. Thymol and carvacrol are the active ingredients of TEO that can be incorporated into the bacterial cell membranes, which would destabilize them^47,48^. Increased permeability eventually causes leakage of significant cellular content and weakening or killing of bacteria in the biofilm. The TEO interferes with the synthesis of extracellular polymeric substances, the protective matrix underlining the stability of biofilms and offering defense mechanisms against most external aggressions. TEO destabilizes the biofilm matrix by reducing the synthesis and accumulation of exopolysaccharides (EPS), rendering the bacteria highly sensitive to antimicrobial agents and immune responses^47^. Moreover, TEO induces intracellular generation of ROS within bacterial cells. This leads to the creation of oxidative stress that can result in damage to all major cellular biomolecules such as proteins, lipids, and nucleic acids. This oxidative damage may impede the formation of biofilms, as well as disperse preformed biofilms^49^. Overall, TEO affects initial adhesion of bacteria to surfaces, the first step that must take place for a biofilm formation. Since TEO prevents bacterial attachment, this decreases its chances of initiating any form of biofilms and further bacterial colonization on any surface. This suggests TEO as a strong natural agent against biofilms, hence representing a valuable tool for avoiding biofilm-related infections and improving conventional antimicrobial treatment.

### Chemical composition of TEO estimated by GC/MS

**Fig. 4:**
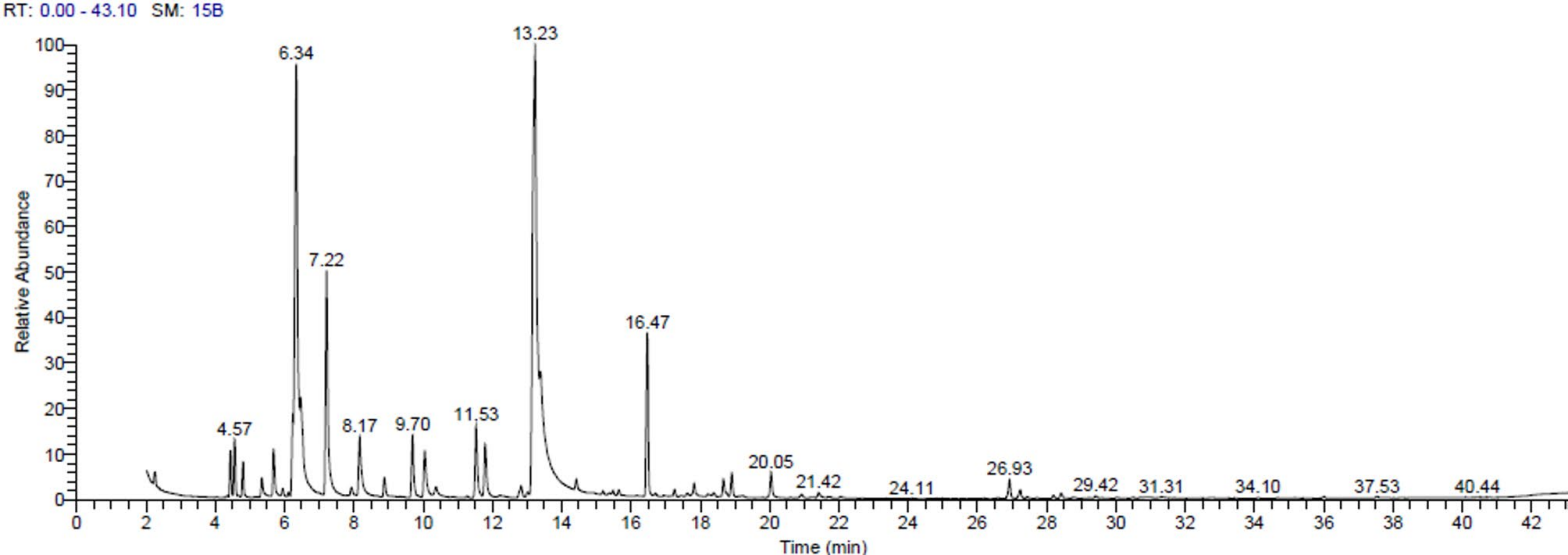
Chromatographic profiles of the thyme essential oil.

The chemical profile of the most prevalent compounds in TEO is illustrated in Fig. 4 and summarized in Table 5. An examination of the primary chemical classes presents in the

**Table 5:** Chemical profile of the most abundant compounds in the thyme essential oil.

Compound	Retention time	Area%	Molecular formula	Molecular wight
Camphene	4.8	1.0	C_10_H_16_	136
α-Thujene	4.44	1.21	C_10_H_16_	136
à-Pinene	4.56	1.52	C_10_H_16_	136
á-Myrcene	5.69	1.55	C_10_H_16_	136
α-Terpinene	6.24	2.06	C_10_H_16_	136
o-Cymene	6.34	18.31	C_10_H_14_	134
Eucalyptol	6.47	5.00	C_10_H_18_O	154
ç-Terpinene	7.22	8.51	C_10_H_16_	136
Linalool	8.16	2.86	C_10_H_18_O	154
endo-Borneol	9.69	2.17	C_10_H_18_O	154
Thymyl methyl ether	11.53	2.64	C_11_H_16_O	164
Thymol	13.24	28.29	C_10_H_14_O	150
Carvacrol	14.43	0.34	C_10_H_14_O	150
Caryophyllene	16.47	5.08	C_15_H_24_	204

EO of thyme plant revealed that TEO was notably rich in oxygenated monoterpenes, with the major compounds; thymol (28.29%), followed by eucalyptol (5%), linalool (2.86%) and borneol (2.17%), then carvacrol (0.34%). Furthermore, TEO exhibited a significant concentration of hydrocarbon monoterpenes. The most prevalent derivatives of this group were o-cymene (18.31%), followed by c-terpinene (8.51%), á-myrcene (1.55%), à-pinene (1.52%) and camphene (1%), which appeared in smaller amounts. In terms of hydrocarbon sesquiterpenes, with caryophyllene being the primary representative accounted for 5.08%. In literature, the fluctuations in the chemical composition of EOs are contingent upon climatic, seasonal, and geographical factors as well as extraction methods and storage time^50^. In the current study, thymol emerged as the predominant volatile component of TEO, which is classified as a phenolic compound exhibiting both antimicrobial and antioxidant properties^26^. Similar studies have documented that the percentage of thymol, which ranges from 12 to 71% for TEO, significantly enhances its antimicrobial efficacy^27,50^. Moreover, thymol and carvacrol are used in agricultural and medical applications considering their multifunctional features. They have not only herbicidal and insecticidal activities, but also, antiviral, antibacterial, and antifungal properties^51^. Similarly, Moo et al.^52^ revealed that caryophyllene can alter membrane permeability and integrity of *B. cereus*, leading to membrane damage, while the results of Guo et al.^53^ proved the efficiency of linalool in damaging the cell wall structure of *P. fluorescens* strain. In the same direction, a variety of *in vitro* and *in vivo* studies have demonstrated the biological properties of camphene, α-thujene, and o-cymene which encompass antibacterial, antifungal, antiviral, anticancer, antioxidant, antiparasitic, antidiabetic, anti-inflammatory effects^54,55^.

### Molecular docking

**Fig. 5:**
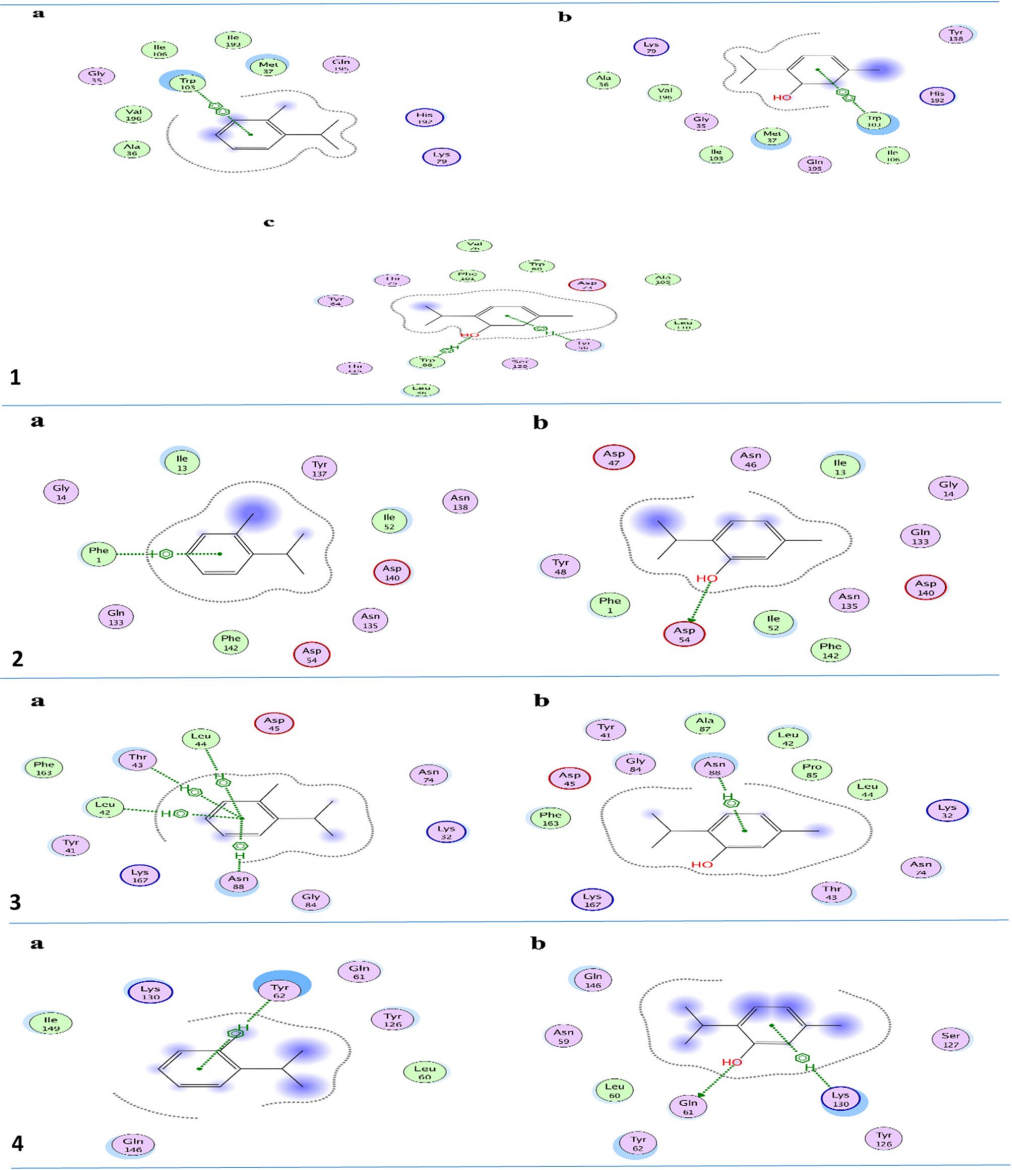
Molecular docking analysis. **1a.** Interaction of thymol against RhlA active site in *P. aeruginosa*. **1b.** Interaction of o-Cymene against RhlA active site in *P. aeruginosa*. **1c.** Interaction of thymol against LasR active site in *P. aeruginosa*. **2a.** Interaction of thymol against FimH active site in *E. coli*. **2b.** Interaction of o-Cymene against FimH active site in *E. coli*. **3a.** Interaction of thymol against ECF active site in *S. aureus*. **3b.** Interaction of o-Cymene against ECF active site in *S. aureus*. **4a.** Interaction of thymol against PrfA active site in *L. monocytogenes*. **4b.** Interaction of o-Cymene against PrfA active site in *L. monocytogenes*.

This study investigated the interactions between key components of TEO, o-cymene, c-terpinene, caryophyllene, and thymol with bacterial proteins important in virulence and biofilm formation using a molecular docking analysis (Fig. 5). Biofilms are structured communities of bacterial cells enveloped in a self-produced polymeric matrix that attaches to surfaces, enhancing bacterial resistance against environmental stresses, including antibiotics^56^. The selected target proteins in this work originate from *P. aeruginosa*, *E. coli*, *S. aureus*, and *L. monocytogenes* are vital to biofilm formation and survival; therefore, they have been strategic targets against biofilms.

RhlA encodes a protein directly involved in the synthesis of rhamnolipids-a class of glycolipids important mainly in biofilm architecture and structural stability. Rhamnolipids confer surface motility, allow the maturation of biofilms, and protect the bacterial cells by forming a barrier against the action of antimicrobial agents^57^. Docking results revealed that only thymol had higher binding affinity for RhlA than o-Cymene since it could form stable hydrogen bonds and π-stacking interactions. The interaction of thymol with RhlA indicates that this compound may reduce the production of rhamnolipids, thus disrupting the biofilm matrix, surface adherence, and resilience of the biofilm to external agents.

Another target in *P. aeruginosa* is LasR, an important regulator in the QS system that coordinates biofilm formation, virulence, and antibiotic resistance according to bacterial population density^58^. QS is an important mechanism in biofilm formation since the processes involved in producing EPS, which forms the matrix of the biofilm, become synchronized. Thymol has demonstrated a tighter binding with LasR and thus may interfere with quorum-sensing signals, a required event in the expression of genes that drive biofilm formation. Any interference at this step will impede the thickness of biofilm formation and overall capability to develop resistance against antibiotics and host defenses.

FimH is a lectin-like adhesin, which is located at the tip of type 1 pili of *E. coli* and is critical for initiating surface attachment, an essential step on the way toward biofilm formation. It mediates adhesion of *E. coli* to host tissues or to inert surfaces, such as urinary catheters, thereby enabling biofilm development at sites, such as the urinary tract. The study presented that thymol is effectively bound to the lectin domain of FimH, especially via hydrogen bonding with key residues. That means, thymol may block FimH-mediated adhesion and, in such a way, interferes with the early events of biofilm formation. This action prevents *E. coli* from establishing chronic infections on surfaces. In this regard, ECF transporter (Energy Coupling Factor) in *S. aureus* is important because it belongs to a family of nutrient uptake systems critical under nutrient-limited conditions, mostly inside host tissues. The ECF transporter would enhance the survival of bacteria through the uptake of B vitamins and other essential nutrients for growth within biofilms. During docking analysis, thymol showed high affinity toward the ECF transporter, thus may interfere with nutrient uptake. This interference could weaken the biofilm through the deprivation of bacterial cells from essential nutrients, thus limiting growth and reducing biofilm resiliency in nutrient-poor environments, such as host tissues or medical devices.

PrfA of *L. monocytogenes* is a transcriptional regulator of virulence genes that plays an important role in cell adhesion, invasion, and intracellular survival^59^. Thymol, through effective binding with PrfA, might downregulate PrfA-regulated genes involved in initiation and maintenance of biofilms and hence weaken *L. monocytogenes* in making strong, symbiotic biofilms, perhaps reducing its persistence on food-contact surfaces. Overall, the results obtained in this docking analysis revealed that only two key components of TEO (thymol and o-cymene) can interact with the binding sites of the tested bacterial proteins. Furthermore, these results suggest that thymol, more than o-cymene, has broad- spectrum potential to inhibit biofilm formation across multiple pathogens by targeting proteins which are important in biofilm development and maintenance. It interferes with key pathways, such as production of rhamnolipids and quorum sensing in *P. aeruginosa*, surface adhesion in *E. coli*, and intake of nutrients in *S. aureus*, and virulence regulation in *L. monocytogenes*; thymol showed a promising anti-biofilm activity. These findings indicate that thymol can act with a natural approach to biofilm inhibition and might, thus, be helpful for inhibiting biofilm-based bacterial infections and persistence on surfaces in clinical and industrial settings.

### Gene expression analysis

**Fig. 6:**
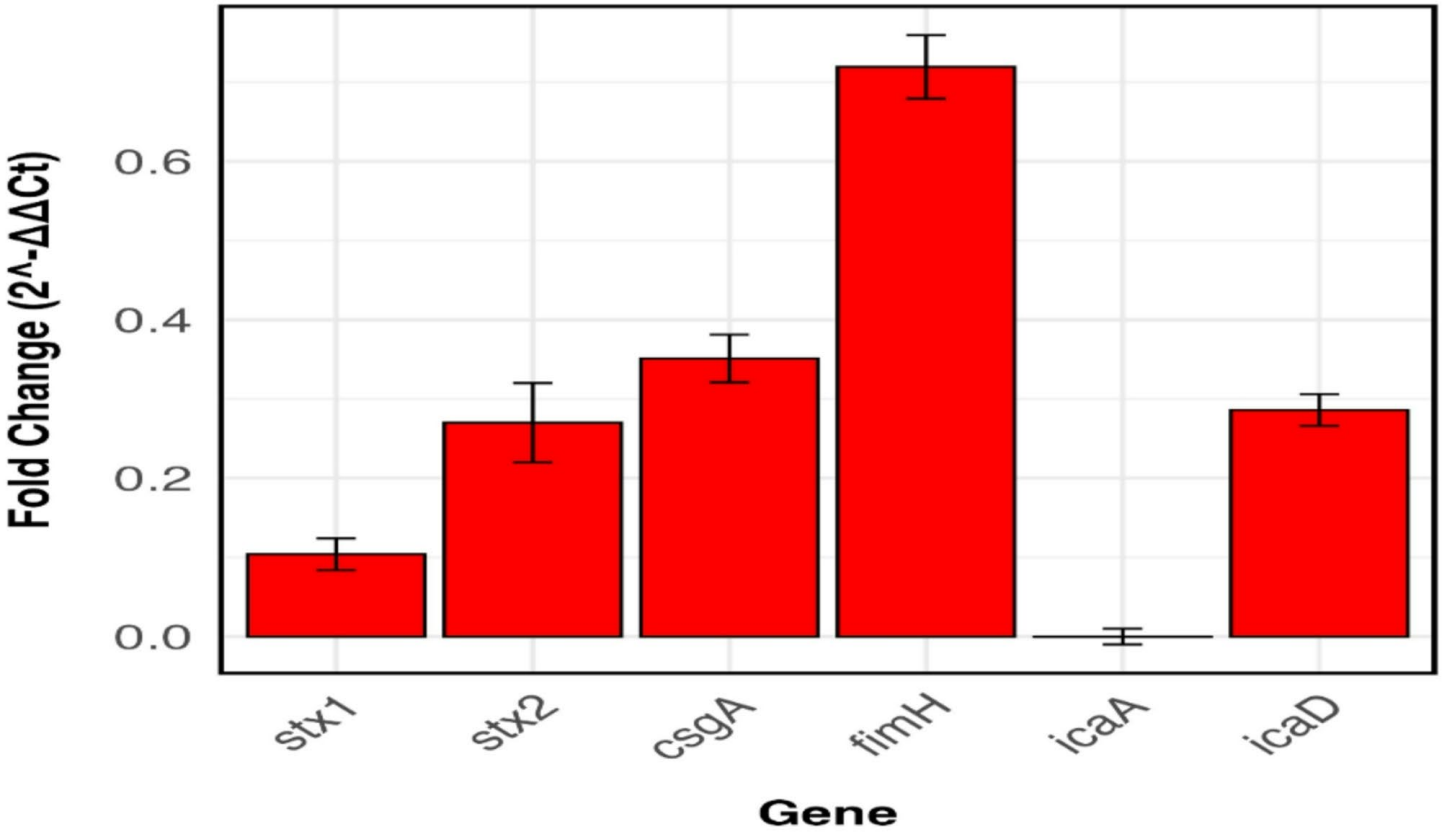
Expression analysis of the virulence and biofilm related genes in *E. coli* O157:H7 and *S. aureus* under control and thyme essential oil treatment conditions. Data represents the mean of three independent replicates. Error bars represent standard deviation.

The differential gene expression of the virulence genes *stx1* and *stx2* in *E. coli* O157:H7 treated with TEO was assessed in comparison to the untreated control. Gene expression analysis revealed a substantial downregulation of both genes in response to the treatment. The fold change in *stx1* expression was 0.104, indicating approximately a 10-fold downregulation in the treatment group. Similarly, *stx2* showed a fold change of 0.270, corresponding to a 3.7-fold decrease compared to the control (Fig. 6). In addition to virulence genes, the expression of *csgA* and *fimH*, which are biofilm-associated genes in *E. coli* O157:H7, was also significantly reduced following treatment. The fold change in *csgA* was 0.410, indicating a 2.4-fold downregulation, while *fimH* exhibited a fold change of 0.450, reflecting a 2.2-fold decrease compared to the control. Similarly, in *S. aureus*, the biofilm-related genes *icaA* and *icaD* showed notable downregulation (Fig. 6). The fold change in *icaA* was calculated as 9.8 × 10^− 8^, indicating a sharped suppression of its expression. Meanwhile, *icaD* exhibited a fold change of 0.620, corresponding to a 1.6-fold decrease in the treatment group. The expression levels of *stx1*, *stx2*, *csgA*, *icaA*, and *icaD* genes in the treatment group were significant (*P* < 0.05) when compared to their expression levels in the untreated control group.

The *shiga* toxin-producing genes *stx1* and *stx2* in *E. coli* O157:H7 are key virulence factors responsible for producing Shiga toxins, which play a critical role in the pathogenicity of this bacterium, particularly in causing severe conditions like hemorrhagic colitis and hemolytic uremic syndrome^60^. These toxins interfere with protein synthesis in host cells, leading to cell damage and inflammation. Essential oils, such as oregano, carvacrol, and clove oil have been shown previously to reduce the expression of *stx1* and *stx2* genes^61,62^. This suppression of Shiga toxin gene expression by essential oils suggests a promising natural strategy to attenuate *E. coli* virulence, potentially limiting the severity of infections and supporting conventional treatments. *csgA* is widely found in members of the Enterobacteriaceae family, particularly in pathogenic *E. coli* strains^63^. CsgA is the primary structural subunit of curli fibers, which form the main component of the extracellular matrix produced by bacteria. This matrix plays a crucial role in reinforcing biofilms, enhancing bacterial resistance to antibiotic treatment, and posing significant challenges to human health^63^. FimH is a key adhesin in *E. coli* that plays a crucial role in biofilm formation and host colonization^64^.

It is a mannose-binding protein located at the tip of type 1 fimbriae, allowing bacteria to adhere to surfaces, including epithelial cells and abiotic structures. This adhesion is the first step in biofilm development, enabling *E. coli* to establish persistent infections, particularly in the urinary tract and medical device-associated infections^22^.

*icaA* and *icaD* genes play a crucial role in biofilm formation in *S. aureus* by encoding enzymes responsible for the synthesis of polysaccharide intercellular adhesin (PIA), a key structural component of the biofilm matrix^65,66^. Their downregulation suggests that TEO effectively disrupts the early stages of biofilm development, potentially limiting the persistence and virulence of *S. aureus*. *Thymus vulgaris* (thyme) and *Cinnamomum verum* (cinnamon) essential oils have been reported previously to exhibit potent antibiofilm activity by modulating the expression of genes crucial for biofilm formation, such as *icaA* and *icaD* genes in *S. aureus*^67^.

## Conclusions

The present study evaluated the significant differences in antibacterial properties among EOs derived from nine distinct herbs. The results revealed that the TEO exhibited vigorous antibacterial efficiency against both Gram-positive and Gram-negative pathogenic bacterial strains as evidenced by the highest inhibition zone values as well as the lowest MIC and MBC values. The TEO possessed bactericidal activities against all tested pathogens, and these findings were confirmed by TEM analysis. Furthermore, TEO induced a complete inhibition of biofilm formation in most of the tested pathogenic bacteria. The chemical profile of TEO demonstrated the presence of highly active compounds (thymol, o-cymene, eucalyptol, carvacrol, terpinene and caryophyllene), which are known for their ability to inhibit bacterial biofilm formation. Generally molecular docking analysis showed that thymol and o-cymene compounds had good interactions with active sites in four tested pathogens. Also, TEO led to the downregulation of virulent and biofilm-formation key genes. The combined evidence from molecular docking, gene expression suppression, and biofilm inhibition demonstrates that TEO is not merely an antimicrobial agent, but a multi-targeted compound with potential to disrupt key bacterial survival and virulence pathways. Thus, these findings highlight its promise as a natural therapeutic candidate in the fight against antibiotic-resistant and biofilm-forming pathogens, with potential applications in the pharmaceutical and food industries.

## Data Availability

The datasets used and/or analysed during the current study available from the corresponding author on reasonable request.

## References

[1] Silbergeld, E. K., Graham, J. & Price, L. B. Industrial food animal production, antimicrobial resistance, and human health. *Annu. Rev. Public. Health*. **29**, 151–169 (2008). 18348709 10.1146/annurev.publhealth.29.020907.090904

[2] Liu, F., Du, L., Zhao, T., Zhao, P. & Doyle, M. P. Effects of phenyllactic acid as sanitizing agent for inactivation of *Listeria monocytogenes* biofilms. *Food Control*. **78**, 72–78 (2017).

[3] Cepas, V. & Soto, S. M. Relationship between virulence and resistance among Gram-Negative bacteria. *Antibiotics* **9**, 719 (2020). 33092201 10.3390/antibiotics9100719 PMC7589547

[4] Sharma, S., Kumar, S., Kumar, P. & Tripathi, V. N. Quorum sensing in Gram-negative pathogens, a fresh look. *Microbe* **4**, 100108 (2024).

[5] Ghavam, M., Bacchetta, G., Castangia, I. & Manca, M. L. Evaluation of the composition and antimicrobial activities of essential oils from four species of *Lamiaceae* Martinov native to Iran. *Sci. Rep.* **12**, 17044 (2022). 36220839 10.1038/s41598-022-21509-5 PMC9553974

[6] Gao, X. et al. Research advances on biogenic amines in traditional fermented foods: emphasis on formation mechanism, detection and control methods. *Food Chem.* **405**, 134911 (2023).

[7] Li, X., Gu, N., Huang, T. Y., Zhong, F. & Peng, G. *Pseudomonas aeruginosa*: A typical biofilm forming pathogen and an emerging but underestimated pathogen in food processing. *Front. Microbiol.* **13**, 1114199 (2023). 36762094 10.3389/fmicb.2022.1114199 PMC9905436

[8] Dhifi, W., Bellili, S., Jazi, S., Bahloul, N. & Mnif, W. Essential oils’ chemical characterization and investigation of some biological activities: A critical review. *Medicines* **3**, 25 (2016). 28930135 10.3390/medicines3040025 PMC5456241

[9] Asante Ampadu, G. A. et al. Antioxidant, antimicrobial, and antibiofilm properties of essential oils extracted from *Dialium guineense*. *Int. J. Food Prop.* **26**, 1885–1902 (2023).

[10] Falleh, H., Ben Jemaa, M., Saada, M. & Ksouri, R. Essential oils: A promising eco-friendly food preservative. *Food Chem.* **330**, 127268 (2020). 32540519 10.1016/j.foodchem.2020.127268

[11] Cáceres, M., Hidalgo, W., Stashenko, E., Torres, R. & Ortiz, C. Essential oils of aromatic plants with antibacterial, Anti-Biofilm and Anti-Quorum sensing activities against pathogenic bacteria. *Antibiotics* **9**, 147 (2020). 32235590 10.3390/antibiotics9040147 PMC7235784

[12] Liu, F. et al. Antibacterial and antibiofilm activities of thyme oil against foodborne multiple antibiotics-resistant *Enterococcus faecalis*. *Poult. Sci.* **99**, 5127–5136 (2020). 32988551 10.1016/j.psj.2020.06.067 PMC7598324

[13] Maggio, F. et al. Anti-biofilm mechanisms of action of essential oils by targeting genes involved in quorum sensing, motility, adhesion, and virulence: A review. *Int. J. Food Microbiol.* **426**, 110874 (2025). 39244811 10.1016/j.ijfoodmicro.2024.110874

[14] Galgano, M. et al. Antimicrobial activity of essential oils evaluated *In vitro* against *Escherichia coli* and *Staphylococcus aureus*. *Antibiotics* **11**, 979 (2022). 35884233 10.3390/antibiotics11070979 PMC9311876

[15] Hassan, A., Rahman, S., Deeba, F. & Mahmud, S. Antimicrobial activity of some plant extracts having hepatoprotective effects. *J. Med. Plants Res.* **3**, 20–23 (2009).

[16] Andrews, J. M. Determination of minimum inhibitory concentrations. *J. Antimicrob. Chemother.* **48**, 5–16 (2001). 11420333 10.1093/jac/48.suppl_1.5

[17] El-Sayed, D., Elsayed, T., Amin, N., Al-Shahaby, A. & Goda, H. Evaluating the phenotypic and genomic characterization of some Egyptian phages infecting Shiga Toxin-Producing *Escherichia coli* O157:H7 for the prospective application in food Bio-Preservation. *Biology* **11**, 1180 (2022). 36009807 10.3390/biology11081180 PMC9404725

[18] Santiago, A. J. et al. Inhibition and dispersal of *Pseudomonas aeruginosa* biofilms by combination treatment with Escapin intermediate products and hydrogen peroxide. *Antimicrob. Agents Chemother.* **60**, 5554–5562 (2016). 27401562 10.1128/AAC.02984-15 PMC4997828

[19] Kyei, L. K., Gasu, E. N., Ampomah, G. B., Mensah, J. O. & Borquaye, L. S. An In Silico Study of the Interactions of Alkaloids from *Cryptolepis sanguinolenta* with Plasmodium falciparum Dihydrofolate Reductase and Dihydroorotate Dehydrogenase. *J. Chem.* 1–26 (2022).

[20] Odoom, J. F. et al. Chemical Composition, Antioxidant, and Antimicrobial Activities of the Leaf and Fruit Essential Oils of the West African Plum, *Vitex doniana*. *J. Chem.* 1–18 (2023).

[21] Wei, Q. & Ma, L. Biofilm matrix and its regulation in *Pseudomonas aeruginosa*. *Int. J. Mol. Sci.* **14**, 20983–21005 (2013). 24145749 10.3390/ijms141020983 PMC3821654

[22] Schembri, M. A. & Klemm, P. Biofilm formation in a hydrodynamic environment by novel FimH variants and ramifications for virulence. *Infect. Immun.* **69**, 1322–1328 (2001). 11179294 10.1128/IAI.69.3.1322-1328.2001 PMC98023

[23] Schleimer, N. et al. The Energy-Coupling factor transporter module ecfaa’t, a novel candidate for the genetic basis of fatty Acid-Auxotrophic Small-Colony variants of *Staphylococcus aureus*. *Front. Microbiol.* **9**, 1863 (2018). 30154773 10.3389/fmicb.2018.01863 PMC6102330

[24] Rouhi, A., Azghandi, M., Mortazavi, S. A., Tabatabaei-Yazdi, F. & Vasiee, A. Exploring the anti-biofilm activity and suppression of virulence genes expression by Thanatin in *Listeria monocytogenes*. *LWT* **199**, 116084 (2024).

[25] Liu, F. et al. Inhibition of biofilm formation and exopolysaccharide synthesis of *Enterococcus faecalis* by phenyllactic acid. *Food Microbiol.* **86**, 103344 (2020). 31703877 10.1016/j.fm.2019.103344

[26] Aljabeili, H. S., Barakat, H. & Abdel-Rahman, H. A. Chemical composition, antibacterial and antioxidant activities of thyme essential oil (*Thymus vulgaris*). *Food Nutr. Sci.* **09**, 433–446 (2018).

[27] Veloso, R. J. et al. Potential of thyme essential oil on arugula sanitization. *Ciênc E Agrotecnol.* **43**, e006819 (2019).

[28] Ozogul, Y. et al. Antimicrobial activity of thyme essential oil nanoemulsions on spoilage bacteria of fish and food-borne pathogens. *Food Biosci.* **36**, 100635 (2020).

[29] Kryvtsova, M. V., Salamon, I., Koscova, J., Bucko, D. & Spivak, M. Antimicrobial, antibiofilm and biochemichal properties of *Thymus vulgaris* essential oil against clinical isolates of opportunistic infections. *Biosyst Divers.* **27**, 270–275 (2019).

[30] Drioiche, A. et al. Correlation between the chemical composition and the antimicrobial properties of seven samples of essential oils of endemic thymes in Morocco against multi-resistant bacteria and pathogenic fungi. *Saudi Pharm. J.* **30**, 1200–1214 (2022). 36164579 10.1016/j.jsps.2022.06.022 PMC9508645

[31] Imelouane, B. et al. Chemical composition and antimicrobial activity of essential oil of thyme (*Thymus vulgaris*) from Eastern Morocco. *Int. J. Agric. Biol* **11**, (2009).

[32] Dorman, H. J. D. & Deans, S. G. Antimicrobial agents from plants: antibacterial activity of plant volatile oils. *J. Appl. Microbiol.* **88**, 308–316 (2000). 10736000 10.1046/j.1365-2672.2000.00969.x

[33] Cristani, M. et al. Interaction of four monoterpenes contained in essential oils with model membranes: implications for their antibacterial activity. *J. Agric. Food Chem.* **55**, 6300–6308 (2007). 17602646 10.1021/jf070094x

[34] Xu, Y., Burton, S., Kim, C. & Sismour, E. Phenolic compounds, antioxidant, and antibacterial properties of pomace extracts from four Virginia-grown grape varieties. *Food Sci. Nutr.* **4**, 125–133 (2016). 26788319 10.1002/fsn3.264 PMC4708636

[35] de Pedro, O. Synthesis, characterization and antifungal activity of quaternary derivatives of Chitosan on *Aspergillus flavus*. *Microbiol. Res.* **168**, 50–55 (2013). 22819383 10.1016/j.micres.2012.06.006

[36] Wang, H. et al. Comparison of phytochemical profiles and health benefits in fiber and oil flaxseeds (*Linum usitatissimum* L). *Food Chem.* **214**, 227–233 (2017). 27507470 10.1016/j.foodchem.2016.07.075

[37] Boskovic, M. et al. Antimicrobial activity of thyme (*Tymus vulgaris*) and oregano (*Origanum vulgare*) essential oils against some Food-borne microorganisms. *Procedia Food Sci.* **5**, 18–21 (2015).

[38] Kang, J., Liu, L., Wu, X., Sun, Y. & Liu, Z. Effect of thyme essential oil against *Bacillus cereus* planktonic growth and biofilm formation. *Appl. Microbiol. Biotechnol.* **102**, 10209–10218 (2018). 30288586 10.1007/s00253-018-9401-y

[39] Dejoies, L., Le Neindre, K., Reissier, S., Felden, B. & Cattoir, V. Distinct expression profiles of regulatory RNAs in the response to biocides in *Staphylococcus aureus* and *Enterococcus faecium*. *Sci. Rep.* **11**, 6892 (2021). 33767282 10.1038/s41598-021-86376-y PMC7994832

[40] Moselhy, M., Abd-Elhafez, K., El-Kholany, E., Gohar, M. & Nasr, N. Antimicrobial, antioxidant and anticancer properties of Globe artichoke and grape by-products as a source of the bio-active phenolic compounds. *Egypt. J. Chem.* **66**, 609–624 (2023).

[41] Madigan, M., Martinko, J. M., Stahl, D. A. & Clark, D. P. Cell structure and function in bacteria and archaea. *In* Brock Biology of Microorganisms. (San Francisco, CA, USA: Pearson Education., USA, (2012).

[42] Da Silva, B. D., Bernardes, P. C., Pinheiro, P. F., Fantuzzi, E. & Roberto, C. D. Chemical composition, extraction sources and action mechanisms of essential oils: natural preservative and limitations of use in meat products. *Meat Sci.* **176**, 108463 (2021). 33640647 10.1016/j.meatsci.2021.108463

[43] Bhavaniramya, S., Vishnupriya, S., Al-Aboody, M. S., Vijayakumar, R. & Baskaran, D. Role of essential oils in food safety: antimicrobial and antioxidant applications. *Grain Oil Sci. Technol.* **2**, 49–55 (2019).

[44] Prasetyoputri, A., Jarrad, A. M., Cooper, M. A. & Blaskovich, M. A. T. The eagle effect and Antibiotic-Induced persistence: two sides of the same coin?? *Trends Microbiol.* **27**, 339–354 (2019). 30448198 10.1016/j.tim.2018.10.007

[45] Guillín, Y., Cáceres, M., Torres, R., Stashenko, E. & Ortiz, C. Effect of Essential Oils on the Inhibition of Biofilm and Quorum Sensing in *Salmonella enteritidis* 13076 and *Salmonella typhimurium* 14028. *Antibiotics* **10**, 1191 (2021). 10.3390/antibiotics10101191 PMC8532617 34680772

[46] Kalia, V. C., Patel, S. K. S. & Lee, J. K. Bacterial biofilm inhibitors: an overview. *Ecotoxicol. Environ. Saf.* **264**, 115389 (2023). 37634478 10.1016/j.ecoenv.2023.115389

[47] Kowalczyk, A., Przychodna, M., Sopata, S., Bodalska, A. & Fecka, I. Thymol and thyme essential Oil—New insights into selected therapeutic applications. *Molecules* **25**, 4125 (2020). 32917001 10.3390/molecules25184125 PMC7571078

[48] Posgay, M., Greff, B., Kapcsándi, V. & Lakatos, E. Effect of *Thymus vulgaris* L. essential oil and thymol on the Microbiological properties of meat and meat products: A review. *Heliyon* **8**, e10812 (2022). 36247140 10.1016/j.heliyon.2022.e10812 PMC9562244

[49] Trabelsi, D., Said, M. B., Hamdane, A. M. & Abdrrabba, M. Application of thyme essential oil for biofilm prevention and water treatment by photosensitization. *Desalin. Water Treat.* **269**, 76–83 (2022).

[50] Sateriale, D. et al. Antibacterial and Antibiofilm Efficacy of Thyme (*Thymus vulgaris* L.) Essential Oil against Foodborne Illness Pathogens, *Salmonella enterica* subsp. *enterica* Serovar *Typhimurium* and *Bacillus cereus*. *Antibiotics* **12**, 485 (2023). 10.3390/antibiotics12030485 PMC10044538 36978352

[51] Özen, İ. et al. Multifaceted applications of thymol/carvacrol-containing polymeric fibrous structures. *Adv. Ind. Eng. Polym. Res.* **7**, 182–200 (2024).

[52] Moo, C. L. et al. Antibacterial activity and mode of action of β-caryophyllene on *Bacillus cereus*. *Pol. J. Microbiol.* **69**, 49–54 (2020). 10.33073/pjm-2020-007 PMC7256763 32162852

[53] Guo, F. et al. Antimicrobial activity and proposed action mechanism of Linalool against *Pseudomonas fluorescens*. *Front. Microbiol.* **12**, 562094 (2021). 33584604 10.3389/fmicb.2021.562094 PMC7875898

[54] Gözcü, S. & Akşi̇T, Z. Chemical composition and antibacterial activity of three volatile oils extracted from Nigella sativa L. Seeds. *Black Sea J. Health Sci.* **6**, 662–666 (2023).

[55] Hachlafi, N. E. *Vitro* and *in vivo* biological investigations of Camphene and its mechanism insights: A review. *Food Rev. Int.* **39**, 1799–1826 (2023).

[56] Shree, P., Singh, C. K., Sodhi, K. K., Surya, J. N. & Singh, D. K. Biofilms: Understanding the structure and contribution towards bacterial resistance in antibiotics. *Med. Microecol*. **16**, 100084 (2023).

[57] Rather, M. A., Gupta, K. & Mandal, M. Microbial biofilm: formation, architecture, antibiotic resistance, and control strategies. *Braz J. Microbiol.* **52**, 1701–1718 (2021). 34558029 10.1007/s42770-021-00624-x PMC8578483

[58] Miranda, S. W., Asfahl, K. L., Dandekar, A. A. & Greenberg, E. P. *Pseudomonas aeruginosa* quorum sensing. *Adv. Exp. Med. Biol.* **1386**, 95–115 (2022). 36258070 10.1007/978-3-031-08491-1_4 PMC9942581

[59] Lemon, K. P., Freitag, N. E. & Kolter, R. The virulence regulator PrfA promotes biofilm formation by *Listeria monocytogenes*. *J. Bacteriol.* **192**, 3969–3976 (2010). 20511507 10.1128/JB.00179-10 PMC2916369

[60] Etcheverría, A. I. & Padola, N. L. Shiga toxin-producing *Escherichia coli*: factors involved in virulence and cattle colonization. *Virulence* **4**, 366–372 (2013). 23624795 10.4161/viru.24642 PMC3714128

[61] Mith, H., Clinquart, A., Zhiri, A., Daube, G. & Delcenserie, V. The impact of oregano (*Origanum heracleoticum*) essential oil and carvacrol on virulence gene transcription by *Escherichia coli* O157:H7. *FEMS Microbiol. Lett.* **362**, 1–7 (2015). 25790499 10.1093/femsle/fnu021

[62] Kim, Y. G. et al. Essential oils and Eugenols inhibit biofilm formation and the virulence of *Escherichia coli* O157:H7. *Sci. Rep.* **6**, 36377 (2016). 27808174 10.1038/srep36377 PMC5093407

[63] Bu, F., Dee, D. R. & Liu, B. Structural insight into *Escherichia coli* CsgA amyloid fibril assembly. *mBio* **15**, e00419–e00424 (2024). 38501920 10.1128/mbio.00419-24 PMC11005368

[64] Knudsen, T. B. & Klemm, P. Probing the receptor recognition site of the FimH adhesin by fimbriae-displayed FimH—FocH hybrids. *Microbiology* **144**, 1919–1929 (1998). 9695925 10.1099/00221287-144-7-1919

[65] Abdel-Shafi, S. et al. The association between IcaA and IcaB genes, antibiotic resistance and biofilm formation in clinical isolates of Staphylococci spp. *Antibiotics* **11**, 389 (2022). 35326851 10.3390/antibiotics11030389 PMC8944761

[66] Jomehzadeh, N. & Emrani, S. S. Assessment of biofilm formation, antibiotic resistance patterns, and the prevalence of adhesion-related genes in clinical *Staphylococcus aureus* isolates. *Heliyon* **11**, e41537 (2025). 39850422 10.1016/j.heliyon.2024.e41537 PMC11755045

[67] Noorbakhsh, F. & Rahmati, P. Effects of thymus vulgaris and cinnamomum verum essential oils on bap and Ica gene expression in *Staphylococcus aureus*. *Arch Clin. Infect. Dis* **17**, (2022).

